# The mass release of migrants from UK immigration detention during the COVID-19 pandemic: what can be learned?

**DOI:** 10.1192/bjb.2021.110

**Published:** 2022-10

**Authors:** Lauren Z. Waterman, Mishka Pillay, Cornelius Katona

**Affiliations:** 1 South London and Maudsley NHS Foundation Trust, London, UK; 2 King's College London, UK; 3 North Central London Clinical Commissioning Group, UK; 4 Royal College of Psychiatrists Working Group on the Health of Refugees and Asylum Seekers, London, UK; 5 One Strong Voice, London, UK; 6 Freedom from Torture, London, UK; 7 Helen Bamber Foundation, University College London, UK

**Keywords:** Asylum seeker, refugee, immigration removal centre, detention, forensic mental health services

## Abstract

Convincing international evidence demonstrates that immigration detention adversely affects mental health. During the COVID-19 outbreak, additional concerns were raised about the safety and appropriateness of immigration detention. Consequently, several hundred migrants were released *en masse* from UK immigration detention centres, and few new detentions took place. Over 70% fewer migrants were held in detention centres in June 2020 compared with December 2019. This large ‘natural experiment’ has demonstrated that detaining fewer migrants is possible and it provides an opportunity to review the necessity for large-scale detention for the purpose of immigration control, as well as its impact on health inequalities. Additionally, given that detainee release arrangements had already been considered unsafe prior to the pandemic, clinicians and service providers should take into consideration that many of those released may not be receiving adequate post-release continuity of care.

The UK detains three times more people in immigration detention centres than any other European country, and this number has risen sharply over the past decade.^1^ The UK is also the only European country with no legal maximum detention period.^2^ Immigration detention is (theoretically) only intended for a small number of specific administrative purposes: to facilitate deportation of migrants, to facilitate the processing of immigration and asylum decisions, and to prevent people from absconding once their immigration application has failed. Immigration detainees include asylum seekers (50%), migrants who have overstayed visas and foreign national ex-prisoners.^1,3^ A minority are detained on arrival, but most after living in the UK for years.^1^ The UK immigration ‘detention estate’ comprises immigration removal centres (IRCs), short-term holding facilities (STHFs) and pre-departure accommodation (PDA). In addition, many people are detained in prisons under immigration powers.

## What is already known about the impact of immigration detention

The mental health of those detained and their access to appropriate assessment and treatment have long been issues of concern. Detainees are from vulnerable groups with a high burden of psychiatric morbidity. Post-migration stressors are also known to be associated with further mental illness and distress.^4^ There is convincing international evidence that immigration detention itself has serious adverse mental health consequences: it precipitates a significant deterioration in mental health in the majority of cases, greatly increasing both the suffering of the individual and the risk of suicide and self-harm.^5,6^ It has also been recognised that IRCs are not appropriate therapeutic environments to promote recovery from mental ill health, owing to the nature of the environment and the lack of specialist mental health treatment resources. Additionally, there is evidence of an association between detention duration and psychiatric morbidity.^13^ Furthermore, findings from longitudinal studies in Australia and the USA suggest that negative effects of detention persist long after release.^7,8^ Therefore, for many years, public bodies, including the British Medical Association (BMA) and many UK medical Royal Colleges, have called for an end to immigration detention. For example, the Royal College of Psychiatrists’ recent Position Statement on the detention of people with mental disorders in IRCs notes that such individuals should be subjected to immigration detention only ‘in very exceptional circumstances’.^9^

Immigration detention also carries a high financial cost to the taxpayer – over £108 million was spent in the financial year 2018–2019, over one-third of the amount spent on overall asylum costs.^10^ However, the Home Office states that detention is necessary in certain cases.^11^

## Post-release continuity of care and health inequalities

A high proportion of detainees are subsequently released into the community,^12^ often without adequate continuity of healthcare or social support. Release may be on a temporary basis and conditional; some migrants are obliged to stay in specified accommodation, be under curfew and/or wear electronic tags. For others, their whereabouts following release is unknown.^13^ Following recent changes to the Immigration Act 2016 and IRC contracts requiring detainees to leave IRC premises within 4 hours of release orders, more are being released suddenly and unpredictably, often into destitution.^12^ This can have a significant impact on their care.

NHS England has identified that release from custody can be a ‘crisis situation’ for some and that it often leads to a worsening of health.^14^ Worryingly, IRC release arrangements have been labelled highly unsafe by clinicians working in the field.^12^

This is particularly concerning since, in the community, asylum seekers, refugees and undocumented migrants are known to experience significant barriers to accessing healthcare.^15–19^ For example, many are unable to advocate sufficiently for themselves and are therefore likely to require inclusion health approaches through targeted action.^17^ Confusion about who is eligible for free care, the effects of upfront charging for services, and the sharing of data between the National Health Service (NHS) and Home Office appear to deter many vulnerable individuals from presenting to services. These individuals often have complex legal situations or are unable to provide the documents requested. Furthermore, NHS administrative staff rarely receive sufficient training in immigration law to determine eligibility for care^18^ and there is often a lack of interpreting services available. A recent study by the Bureau of Investigative Journalism found that less than a quarter of GP surgeries (24%) surveyed across England, Scotland and Wales would register someone without proof of address, proof of ID or legal immigration status, despite NHS and Royal College of General Practitioner guideance that this is an unacceptable reason to deny someone registration.^19^

Difficulties with healthcare access have been further exposed and exacerbated during the COVID-19 pandemic, which appears to have widened the health inequality gap for vulnerable groups. According to a recent report by Medact, many asylum seekers and undocumented migrants have not sought healthcare during the pandemic owing to fear and mistrust in the context of the government's wider hostile environment policies, such as NHS charging and data sharing with the Home Office. This has remained the case despite the COVID-19 exemption from charging and has deterred many of those who are in fact entitled to free healthcare. Additional barriers during the pandemic include not having access to technical devices for remote consultations, problems with housing and distance from care services. Some have avoided healthcare owing to fears of contracting COVID-19, exacerbated by fears of discriminatory treatment and the disproportionate number of deaths among Black, Asian and minority ethnic (BAME) communities.^20^ As the majority of people with irregular immigration statuses are from BAME communities, they are at an increased risk of contracting COVID-19, in addition to experiencing more severe COVID-19-related symptoms and higher death rates.^21–23^ There is also evidence of specific mental health effects relating to COVID-19 that disproportionately affect people from these communities and require specialised input, and are likely to persist long after the pandemic has ended.^24^

## Changes to immigration detention practices during the COVID-19 outbreak

During the first few months of the COVID-19 outbreak, additional concerns were raised about the safety and appropriateness of immigration detention. Indeed, the first-tier tribunal granted bail to immigration detainees on the grounds that they could not be returned to their country of origin or have their immigration applications processed promptly during the outbreak, rendering their incarceration unlawful.^25^ They also took into consideration public health concerns about COVID-19 outbreaks in detention centres where social distancing was unfeasible.^26^ Consequently, over 700 migrants, including asylum seekers, were released *en masse* from UK immigration detention centres. Hardly any people were newly detained during this time.

**Fig. 1:**
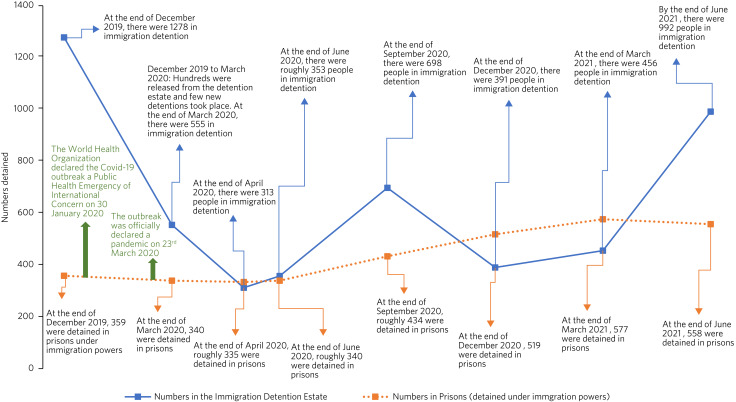
Timeline of changes to the number of migrants detained in the UK under immigration powers during the COVID-19 outbreak.

Statistics are published quarterly by the Home Office on the numbers of people held in the detention estate,^27^ and an additional COVID-19-related publication provides some statistics on the numbers detained between March 2020 and the start of May 2020.^28^ In addition to these, the Home Office publishes statistics on the numbers of people held in prisons under Immigration Act powers. According to these statistics, 70% fewer migrants were in detention centres in June 2020 compared with December 2019. Following the easing of pandemic-related restrictions in Spring 2021, the numbers detained rose again sharply. The number of people detained in prisons under immigration powers has remained relatively stable. The changes in the number of migrants in detention during the COVID-19 outbreak are summarised in the timeline in Fig. 1.

The UK was not the only country that released immigration detainees *en masse*. For example, within a month of the start of the pandemic in March 2020, Spain had emptied out its detention centres, leaving just three people detained under immigration laws in mainland Spain. This process resulted from a collaboration between local and regional authorities cooperating with civil society organisations, the Ombudsman, detention centre directors and judges to ensure that the rights and dignity of migrants were respected. Partial and large-scale releases also took place in other countries, including in The Netherlands, Indonesia, France, Peru, Thailand and the USA.^29^

## A ‘natural experiment’ has been created

As shown above, the number of people in immigration detention significantly reduced during the earlier stages of the COVID-19 outbreak, when people were released from immigration detention *en masse* and few new detentions took place. Based on the recent figures, detention practices appear to be returning to how they were pre-pandemic. These unusual circumstances have created a ‘natural experiment’, providing a new lens with which to examine and learn from detention practices.

Natural experiments have a long history in public health research and are seen as key in reducing health inequalities by enabling real-world evaluation of the impact of environmental changes and health interventions. A natural experiment is an empirical study in which individuals are exposed to experimental and control conditions that are determined by nature or by other factors outside the control of the investigators. As such, they usually rely on existing (including routinely collected) data. They differ from observational studies in that they include a comparison of conditions that enable inferences to be made about causation.^30,31^ In this case, the control group would be migrants who were detained in immigration detention prior to the COVID-19 outbreak, and the experimental group would be migrants who, during the COVID-19 outbreak, would have been subject to immigration detention but were not detained owing to these changes resulting from the outbreak.

These conditions provide a unique opportunity for the Home Office and healthcare providers to look at the results of this natural experiment, by examining the immigration consequences and health outcomes from these changes. What is already clear from the COVID-19 experience is that detaining fewer people is ‘possible’. Furthermore, the partial suspension of immigration detention in the UK (and other countries, such as Spain) has not (as far as we are aware) been reported to have had any immediate negative consequences. This raises the fundamental question of whether detention (at least on the scale practised in the UK prior to the COVID-19 pandemic) is a necessary means of immigration control; or, alternatively, whether community-focused approaches to hosting migrants are sufficient, while also being cheaper and more humane, facilitating integration into society, and being less harmful to physical and mental health.

## A pressing need for further data

The Home Office has stated that it is committed to fixing a broken asylum system and creating a fairer system,^11^ although its recent Nationality and Borders Bill has been condemned by national and international bodies, who contested that it is explicit in its intention to punish refugees who seek safety in the UK.^32,33^ We therefore urge the Home Office to make full use of this natural experiment to inform decisions about the future of detention within the fairer asylum system that they have stated they are committed to developing. They should use the natural experiment to review critically whether detention has been fulfilling its intended objectives sufficiently to justify its ongoing use despite its known health, moral and financial costs, and the necessity of continuing to detain people in immigration detention centres. For example, we propose that the Home Office should, collaboratively with relevant experts such as health economic experts and healthcare professionals, conduct a study on the health economics of current immigration detention practices compared with detaining fewer people and for less time. Further data regarding the health outcomes of those released are also urgently required.

There have previously been calls for governments to invest and capitalise on data sources such as these, including the infrastructure for linking exposure and outcome data, so that valuable conclusions can be drawn to influence policy and practice.^30^ So far, the data collected and made publicly available by governmental departments such as the Home Office relating to descriptive statistics and the health and social situation of this population have been limited, leaving considerable gaps in the data we currently have access to. This is even more problematic during the current COVID-19 outbreak, where health inequalities appear to be having a greater and more severe impact on this population.

If, in fact, detention is shown not to be necessary as a form of immigration control, with community-based methods sufficient for this objective, then the purpose of continuing with immigration detention would surely only be to contribute to the UK's ‘hostile environment’^34^ to deter people from immigrating. This would be unethical in light of the clear negative impact on those detained.

## Concerns over post-release care – how healthcare professionals can help

As highlighted above, release arrangements for detainees prior to the pandemic were already considered highly unsafe. Therefore, there is also a specific concern that most of the hundreds of migrants released during the pandemic did not have adequate continuity of care plans arranged, particularly given the sudden nature of their release. This is of relevance to clinicians supporting them in the community, who may encounter people who have previously been detained during their clinical practice. For example, it is likely to be up to those clinicians to help previously detained migrants to access healthcare, medications and non-governmental organisation (NGO) support – vulnerable migrants may not otherwise receive this support and find themselves unable to navigate the complex healthcare environment. Additionally, an opportunity is being missed to follow up these individuals to find out what happened to them, where they went, how their health changed and how they accessed healthcare. Box 1 outlines some ways in which UK healthcare professionals can help.

Box 1 How UK healthcare professionals can help: practical pointers

- Familiarise yourselves with the legal entitlements to NHS healthcare for migrants in the UK and common barriers to accessing care (see the *BJPsych Bulletin* article ‘Assessing asylum seekers, refugees and undocumented migrants’)^35^
- Help previously detained migrants to register with a GP and remind GP practices of NHS England's guidance that patients must not be refused registration on the grounds that they are not able to provide proof of address, identification or immigration status^36^
- Help them to access free prescriptions (via an HC2 certificate in the UK)
- Ensure that they are receiving repeat prescriptions for regular medication
- Signpost them to relevant NGOs that support migrants
- Be aware of the potentially traumatic impact that immigration detention can have on people and what psychological support they may benefit from
- Campaign with charities such as Doctors of the World, Detention Action or one of Medact's ‘Patients not passports’ groups

## Conclusions

Immigration detention is already known to carry a high financial and moral cost and to severely affect the mental health of those detained. The health impact of immigration detention is of interest to the wider medical community who advocate for their patients: national bodies, including the BMA and medical Royal Colleges, have been campaigning against its use owing to adverse consequences for detainees’ health. The medical community must continue to put pressure on the Home Office to collect and review the necessary data to ensure that its immigration practices are backed up by evidence (including using the data created by the natural experiment described in this article), to review the effects of releasing migrants from detention without adequate continuity of care as well as the need for immigration detention in the first place.

## Data Availability

Data availability is not applicable to this article as no new data were created or analysed in this study.

## About the authors

**Lauren Waterman** is a Specialty Trainee Year 6 (ST6) in psychiatry and an Academic Clinical Fellow at South London and Maudsley NHS Foundation Trust. She is also a Mental Health Research UK funded MD(Res) doctoral student at King's College London, a Health Education England Population Health Fellow with North Central London Clinical Commissioning Group and a Member of the Royal College of Psychiatrists’ Working Group on the Health of Refugees and Asylum Seekers, London, UK. **Mishka Pillay** is an expert by experience campaigner at One Strong Voice, a member of the Royal College of Psychiatrists’ Working Group on the Health of Refugees and Asylum Seekers, London, and a trustee at Freedom from Torture, UK. **Cornelius Katona** is Medical and Research Director of the Helen Bamber Foundation, London, and Honorary Professor in the Division of Psychiatry, University College London.
